# A Twitter dataset for Monkeypox, May 2022^☆^

**DOI:** 10.1016/j.dib.2023.109118

**Published:** 2023-04-14

**Authors:** Zahra M. Nia, Nicola L. Bragazzi, Jianhong Wu, Jude D. Kong

**Affiliations:** a Africa-Canada Artificial Intelligence and Data Innovation Consortium (ACADIC), York University, Canada; b Laboratory for Industrial and Applied Mathematics (LIAM), York University, Canada

**Keywords:** Monkeypox, Pandemic, Social media, Twitter developers API

## Abstract

After struggling with COVID-19 pandemic for two years, the world is finally recovering from this crisis. Nonetheless, another virus, Monkeypox, is quickly spreading throughout the world and in non-endemic regions and continents, threatening the world to a new pandemic. Twitter as a popular social media has successfully been used for predicting and controlling outbreaks. Much research previously has been done for building early warning systems, trend prediction, and misinformation and fake news detection. Since tweets are not accessible to all researchers, in this work, a publicly available dataset containing 2400202 tweets gathered from May first to December twenty-fifth, 2022 is presented. Twitter developers academic researcher API which returns all the tweets matching a given query was used to gather the dataset. To this end, the full archive search and keywords related to Monkeypox and its equivalents in other languages, i.e. Monkeypox or “monkey pox” or “viruela dei mono” or “variole du singe” or “variola do macoco” were used. The retweets were excluded using the negation operator, and the tweet ids and user ids were extracted and shared with public. Approximately, 1.79 percent (43047 number) of tweets were geotagged. To visualize the geotagged tweets, the longitude and latitude of the bounding box coordinates were averaged. This work will help researchers shed light on the news, patterns, and on-going discussions of Monkeypox on social media, identify hotspots, and help contain the Monkeypox virus.

**Specifications Table**

**Table:** 

Subject:	Health and medical science, Infectious Diseases
Specific subject area:	This dataset contains tweets related to current Monkeypox outbreak. It is primarily released to help researchers contain the outbreak. Basically it is classified under Health and medical science, Infectious Diseases. But it can also be useful to scientists from areas such as Data Science, Computer Science, Social Science, Mathematics and Statistics, and even Economy.
Type of data:	Table
How the data were acquired:	This data was gathered using Twitter developer's academic researcher API. The full archive search endpoint that returns all the tweets available with a certain query was used to gather all the tweets, except the retweets gathered with keywords Monkeypox or “monkey pox” or “viruela dei mono” or “variole du singe” or “variola do macoco”, from May first to December twenty-fifth, 2022. A number of 2400202 tweet ids and user ids were shared with the public.
Data format:	Raw Filtered (Retweets are excluded)
Description of data collection:	One limitation to this dataset is that it was gathered from May first to December twenty-fifth 2022. Tweets posted in the future cannot be included in this dataset. Another limitation is that due to Twitter developers’ privacy policy agreement only tweet ids and user ids can be shared with the public. To acquire the actual tweets and other metadata the tweets ids need to be hydrated.
Data source location:	The dataset includes all the geotagged and non-geotagged tweets posted in any language from any country and location.
Data accessibility:	The dataset is available at Mendeley: Nia, Zahra; Bragazzi, Nicola; Kong, Jude; Wu, Jianhong (2022), “A Twitter Dataset for Monkeypox, May-Dec, 2022”, Mendeley Data, V1, doi: 10.17632/242whtdt3m.1 Repository name: Monkeypox_May1_to_Dec25_2022.csv Direct URL to data: https://doi.org/10.17632/242whtdt3m.1 And Dryad: Repository name: Monkeypox_May1_to_Dec25_2022.csv Direct URL to data: https://doi.org/10.5061/dryad.zpc866tbh The dataset includes only tweet ids and user ids in compliance with Twitter developer's term of use and privacy policy [1]. To retrieve the actual tweets and other metadata, create data, number of retweets, number of likes, etc, the tweet ids have to be hydrated. DocNow is one user-friendly software that hydrates tweet ids [2]. After installation, DocNow should be authorized using the Twitter API key generated for your Twitter developer's account. Next, the file containing the tweet ids is uploaded to the software. By default, the tweets and their metadata are returned in .json. However, it can be set to return in other formats such as .csv, as well [3].

## Value of the Data

• The COVID-19 pandemic has created havoc throughout the world. After more than two years, just when the Non-Pharmaceutical Interventions (NPI) are being lifted, and the world needs to recover from the damages caused, a new virus, Monkeypox, emerges in more than 20 countries, and threatens the globe to a new pandemic.
• NPIs have canceled or postponed many surgeries, diagnostic tests (e.g. cancer, MRI, and CT scans) and procedures (e.g. orthoptics, pediatrics, and dentals), causing a great number of patients to fall out of their timeline [4]. Moreover, the number of patients from chronic diseases such as diabetes, hypertension, and cardiovascular disease have increased [5,6]. Mental health disorder has escalated in adults, as well as children and adolescents, especially in healthcare workers [7], [8], [9]. Worst of all, global economy is facing a recession, substantially in lower and lower-middle income countries [10]. The world cannot bear another catastrophe.
• It is critical to contain the Monkeypox virus and extinguish the menace. Twitter has previously been successful in early warning systems for outbreaks [11], trend prediction [12], hotspot identification [13], and misinformation and fake news detection [14]. This dataset could help researchers advance studies concerning Monkeypox and provide further insights to bring the outbreak under control [15].
• Researchers from Data Science, Computer Science, Social Science, Mathematics and Statistics, Medicine and even Economy can use Twitter data further to understanding misinformation/disinformation regarding Monkeypox [16], stigmatization of Africans and LGBTQ+ for spreading Monkeypox [17], understanding topics of public concern regarding Monkeypox [18], and predicting the trends of Monkeypox [19].
• The results of the studies could be used by decision-makers to inform more targeted policies, and health officials to provide better services suitable for all communities especially vulnerable and marginalized populations.
• Social media platforms such as Twitter are increasingly being used by public to discuss their opinions, concerns, and experiences. This dataset could help researchers understand the popularity of Twitter posts over time, locations and hotspots where people are more concerned, the discussed topics at their hotspots, and sentiments/emotions of the topics of concern.
• Previously, a Twitter dataset was prepared for Monkeypox in June 2022 [20]. However, the dataset includes 68934 tweets and is gathered with RapidMiner [21], not Twitter API, and does not include all the tweets available with the utilized keywords. This dataset includes 2400202 tweets gathered with a Twitter API academic researcher account that contains all the tweets available with the keywords used from May 1 to December 25, 2022. Thus, it could provide better insights on popular discussion and help studies regarding Monkeypox concerns be less prone to error.

## 1 Data Description

Each line in the file Monkeypox_May1_to_Dec25_2022.csv is associated with a defferent tweet and includes two columns, TweetID and AuthorID which represent the tweet id and the user id. The file includes 2400202 lines in total. To access the actual tweets and their metadata, the tweet ids need to be hydrated. One software that can hydrate the tweets is DocNow hydrator [2]. After installing the software, in order to use it, one must have a Twitter account. Using your Twitter account, you get a Twitter API key that is used to authorize the hydrator. When the hydrator is authorized, the file containing the tweet ids is feed into it. In the add tab the “select Tweet ID File” should be selected to upload the file. Next, a name is set for the hydrator file and “Add dataset” is clicked. Finally, by clicking on start button the hydration process begins. The files are saved in a .json file by default. However, it is possible to save the files in .csv format as well [3].

The tweets belong to 69 different languages. Roughly, 81.82 percent (1963797 number) of the tweets are in English. Table 1 presents the ten languages that include a higher portion of the tweets with examples.

**Table 1:** The portion of the tweets belonging to each language with examples.

**Fig. 1:**
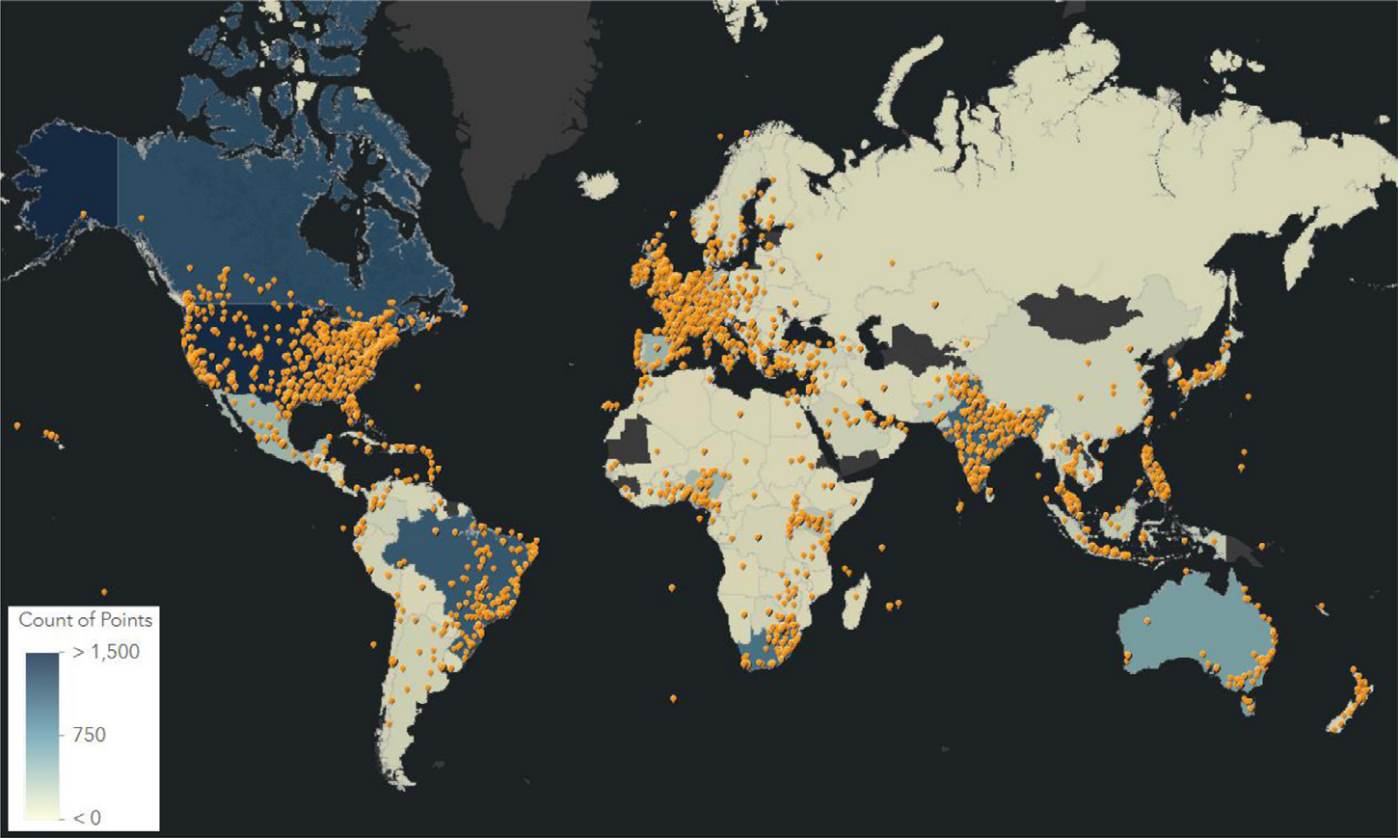
Visualization of geotagged tweets.

About 1.79 percent (43047 number) of tweets are geotagged. The longitude and latitude of geotagged tweets were estimated by averaging the longitude and latitude of their bounding box coordinates. Fig. 1 which was created using ArcGis Online visualizes the location of the tweets. Approximately, 1.03 percent (24650) of the geotagged tweets were from the United States. Table 2 shows the ten countries which had the highest percentage of the geotagged tweets. More information on the geotagged tweets is available at [22].

**Table 2:** The portion of the tweets belonging to each country.

Country	Percentage of Tweets	Number of Tweets
The United States	1.03	24650
The United Kingdom	0.15	3563
Canada	0.08	1967
France	0.06	1378
Brazil	0.05	1257
India	0.04	1066
South Africa	0.04	1015
Republic of Philippines	0.03	729
Australia	0.02	581
Spain	0.02	536
Other	98.48	2363458

Twitter as one of the most popular social media platforms is capable of providing researchers with information to understand the global situation better, and help reduce the number of cases. Therefore, in this work, a dataset containing all the tweets posted since May first to December twenty-fifth 2022 is presented. This dataset can be updated in the future and help researchers overcome various issues regarding the current Monkeypox outbreak.

## 2 Experimental Design, Materials and Methods

Twitter API academic researcher account returns all the tweets available with a certain query and allows the user to retrieve ten million tweets per month. The full archive search of the Twitter API academic researcher account was used to retrieve the tweets. This endpoint accepts a query as input which includes a set of keywords and returns all the tweets and their metadata that match the keywords. Since European countries are the hotspots for current Monkeypox virus, the keywords used to build the query included Monkeypox and its equivalents in Spanish, French, and Romanian, i.e., Monkeypox or “monkey pox” or “viruela dei mono” or “variole du singe” or “variola do macoco”. In addition, the retweets were excluded using the negation operator, -is:retweet. The tweets were gathered from May first to December twenty-fifth 2022, and 2400202 number of tweets were retrieved. Other than the actual text, the metadata obtained included tweet id, conversation id, in reply to user id and in reply to username (in case of the tweet being a reply), created at, type (i.e. tweet, replied to, or quoted), language, retweets count, reply count, like count, geo id, geo-country, geo-province/city, geo-coordinates, author id, author name, author username, author description, author-reported location, author hashtags, created account at, follower count, following count, tweet count, and image URL. However, due to Twitter developers’ privacy policy agreement, only the tweet ids and user ids are shared with the public [1]. Therefore, in order to use the dataset, the tweets need to be hydrated [3]. Our dataset includes all the geotagged and non-geotagged tweets posted in any language and from any country.

## Ethics Statements

This dataset complies with the Twitter developers’ API terms of use and privacy policy [1].

## CRediT authorship contribution statement

**Zahra M. Nia:** Conceptualization, Data curation, Formal analysis, Investigation, Methodology, Validation, Visualization, Writing – original draft, Writing – review & editing. **Nicola L. Bragazzi:** Conceptualization, Formal analysis, Investigation, Methodology, Validation, Supervision, Writing – original draft, Writing – review & editing. **Jianhong Wu:** Conceptualization, Formal analysis, Funding acquisition, Investigation, Methodology, Resources, Supervision, Software, Validation, Writing – review & editing. **Jude D. Kong:** Conceptualization, Formal analysis, Funding acquisition, Investigation, Methodology, Project administration, Resources, Software, Supervision, Validation, Writing – review & editing.

## Declaration of Competing Interest

The authors declare that they have no known competing financial interests or personal relationships that could have appeared to influence the work reported in this paper.

## Data Availability

A Twitter Dataset for Monkeypox, May-Dec, 2022 (Reference data) (Mendeley Data).
